# Association between maternal nutritional status in pregnancy and offspring cognitive function during childhood and adolescence; a systematic review

**DOI:** 10.1186/s12884-016-1011-z

**Published:** 2016-08-12

**Authors:** Sargoor R. Veena, Catharine R. Gale, Ghattu V. Krishnaveni, Sarah H Kehoe, Krishnamachari Srinivasan, Caroline HD Fall

**Affiliations:** 1 Epidemiology Research Unit, CSI Holdsworth Memorial Hospital, Mysore, India; 2 MRC Lifecourse Epidemiology Unit, University of Southampton, Southampton, UK; 3 Department of Psychology, Centre for Cognitive Ageing and Cognitive Epidemiology, University of Edinburgh, Edinburgh, UK; 4 St. John’s Research Institute, St. John’s National Academy of Health Sciences, Bangalore, India

**Keywords:** Maternal adiposity, Maternal micronutrients, Pregnancy, Children, Cognition

## Abstract

**Background:**

The mother is the only source of nutrition for fetal growth including brain development. Maternal nutritional status (anthropometry, macro- and micro-nutrients) before and/or during pregnancy is therefore a potential predictor of offspring cognitive function. The relationship of maternal nutrition to offspring cognitive function is unclear. This review aims to assess existing evidence linking maternal nutritional status with offspring cognitive function.

**Methods:**

Exposures considered were maternal BMI, height and weight, micronutrient status (vitamins D, B12, folate and iron) and macronutrient intakes (carbohydrate, protein and fat). The outcome was any measure of cognitive function in children aged <18 years. We considered observational studies and trials with allocation groups that differed by single nutrients. We searched Medline/PubMed and the Cochrane Library databases and reference lists of retrieved literature. Two reviewers independently extracted data from relevant articles. We used methods recommended by the Centre for Reviews and Dissemination, University of York and the Preferred Reporting Items for Systematic Reviews and Meta-Analyses (PRISMA) statement.

**Results:**

Of 16,143 articles identified, 38 met inclusion criteria. Most studies were observational, and from high-income settings. There were few randomized controlled trials. There was consistent evidence linking maternal obesity with lower cognitive function in children; low maternal BMI has been inadequately studied. Among three studies of maternal vitamin D status, two showed lower cognitive function in children of deficient mothers. One trial of folic acid supplementation showed no effects on the children’s cognitive function and evidence from 13 observational studies was mixed. Among seven studies of maternal vitamin B12 status, most showed no association, though two studies in highly deficient populations suggested a possible effect. Four out of six observational studies and two trials (including one in an Iron deficient population) found no association of maternal iron status with offspring cognitive function. One trial of maternal carbohydrate/protein supplementation showed no effects on offspring cognitive function.

**Conclusions:**

Current evidence that maternal nutritional status during pregnancy as defined by BMI, single micronutrient studies, or macronutrient intakes influences offspring cognitive function is inconclusive. There is a need for more trials especially in populations with high rates of maternal undernutrition.

**Systematic review registration:**

Registered in PROSPERO CRD42013005702.

**Electronic supplementary material:**

The online version of this article (doi:10.1186/s12884-016-1011-z) contains supplementary material, which is available to authorized users.

## Background

Policy makers and health professionals worldwide recommend a nutritious diet for pregnant mothers to ensure a healthy pregnancy. The mother’s diet and nutrient stores are the only source of nutrition for the growing fetus, and are likely to influence offspring neurodevelopment, which occurs rapidly during the intrauterine period [1]. While macronutrients (carbohydrate, protein and fat) serve as building blocks in overall brain development, micronutrients, including vitamins and minerals enable myelination, synaptogenesis, neurotransmitter production and transmission [1].

Three systematic reviews on maternal nutrition and offspring cognitive function were published in 2011. One examined the association of pre-pregnancy and pregnancy obesity with offspring neurodevelopmental outcomes. It included 12 observational studies, of which only two investigated cognitive function. It concluded that children of obese women may be at increased risk of cognitive deficits [2]. The second examined the effect of prenatal folic acid supplementation with other vitamins/minerals on childhood mental performance. It included only two studies, both randomized controlled trials (RCT), in which cognitive function was measured in children during infancy and childhood. It concluded that prenatal multivitamin supplements containing folic acid do not affect the child’s cognitive function [3]. The third review evaluated 18 RCT’s to assess evidence for beneficial effects of single or multiple micronutrient supplementation (vitamins, minerals, fatty acids, and protein and carbohydrate in different combinations) during pregnancy on offspring cognitive and/or behavioural outcomes during infancy and early childhood [4]. Out of 18 studies 17 assessed offspring cognitive function. Among them, two studies used zinc supplementation alone, one used iron supplementation alone, eight used foods rich in n-3 fatty acids as supplements and six used multiple micronutrient supplements. The review found evidence for a beneficial effect of maternal n-3 fatty acids (4 out of 8 studies) and multiple micronutrient supplementation (3 out of 6 studies) but no evidence of benefit from zinc or iron supplementation alone. The authors concluded that the evidence was inconclusive due to transient findings, methodological limitations and inadequate reporting and suggested further research. As new data have been published since these reviews a new review is warranted.

This systematic review focusses on studies linking cognitive function in children to the following indices of maternal nutritional status: a) anthropometry (body mass index (BMI), height and weight); b) status or intake of selected single micronutrients (vitamins D, B1, B6, B12 and folate) and iron and c) dietary intake of macronutrients (carbohydrate, protein and fat). It does not cover trials of multiple micronutrient, or fatty acids, which have been adequately covered in recent reviews.

## Methods

We used the methods recommended by the Centre for Reviews and Dissemination (CRD), University of York [5] and followed the Preferred Reporting Items for Systematic Reviews and Meta-Analyses (PRISMA) statement [6].

### Eligibility criteria, search strategy and identification of literature

Our exposure of interest was maternal nutritional status during pregnancy: BMI, height and weight; status or intake of selected single micronutrients (vitamins D, B1, B6, B12 and folate) and iron and dietary intake of macronutrients (carbohydrate, protein and fat). Our outcome was any measure of cognitive function in children aged <18 years. We included observational studies and trials, published in English from January 1960 to October 2014, and excluded case reports and animal studies. We searched Medline/PubMed and the Cochrane Library using the medical subject headings (MeSH) terms and text word terms shown in Table 1. A lateral search (screening of reference lists of literature retrieved for review) was carried out.

**Table 1:** List of MeSH terms and the text word terms used for exposure and outcome

Exposure: Maternal nutritional status during pregnancy		Outcome: Childhood and adolescent cognitive function	
MeSH terms	Text word terms	MeSH terms	Text word terms
“exp body weight/or exp body mass index/or exp anthropometry/or exp body size/or exp skinfold thickness/or exp nutrition assessment/or exp nutritional status/or exp mothers/or exp pregnancy/or exp malnutrition/or exp diet vegetarian/or exp haemoglobin/or pregnancy complications/or exp anemia/or exp folic acid/or exp folic acid deficiency/or exp vitamin b12 deficiency/or exp ferritin/or exp iron, dietary/or exp cholecalciferol/or exp pyridoxine/or exp vitamin b complex/or exp riboflavin/or exp thiamine/or exp vitamin D/”	“maternal nutrition or maternal anthropometry or pregnancy nutrition or antenatal nutrition or intrauterine nutrition or gestational nutrition or maternal undernutrition or prenatal nutrition or maternal BMI or maternal micronutrients or vegan mothers or vegetarian mothers or macrobiotic mothers or maternal folate or maternal folic acid or maternal vitamin b12 or maternal cobalamin or maternal vitamin D or 25 hydoxy vitamin D or maternal cholecaliciferol or maternal haemoglobin or maternal iron or maternal B vitamins or maternal vitamin b1 or maternal vitamin b6 or maternal vitamin b9 or maternal b vitamins or maternal anaemia or maternal diet”	“exp child/or exp child development/or exp adolescent/or exp neurobehavioral manifestations/or exp child, preschool/or exp cognition, physiology/or exp attention/or exp memory, long-term/or exp memory, short-term/or exp memory/or exp intelligence tests/or exp psycho motor performance/or exp child psychology/or exp decision making/or exp psychometrics/or exp intelligence/or exp mental competence/or exp cognition/or exp motor skills/or exp language development/or exp learning/or exp verbal learning/or exp problem solving/or exp perception/or exp thinking/or exp executive function/or exp function/or exp human development/or exp adolescent development/or exp speech/or exp mental processes/	Cognitive function or intelligence or IQ or executive function or psychomotor development or cognitive performance or cognition or educational attainment or cognitive ability or cognitive deficits or intellectual ability or learning or memory or language development.

*MeSH* Medical subject headings

We included trials if they used a single micronutrient or if it was a multiple micronutrient trial which included intervention groups that differed by a single micronutrient. We did not assess the effects of multiple micronutrient supplements or fatty acids which have been the subject of recent systematic reviews.

**Fig. 1:**
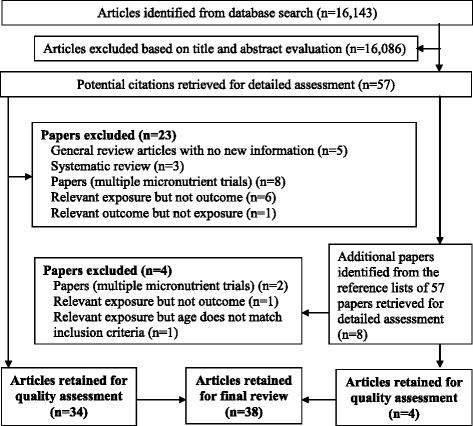
Flow diagram illustrating the selection of literature for inclusion in the qualitative synthesis

From the database search 16,143 articles were identified (Fig. 1). Their titles and abstracts were evaluated, and 57 were eligible. Another 8 articles were identified by lateral search, making a total of 65 for full review. 27 were excluded leaving 38 studies for final evaluation.

### Data extraction and quality assessment

Data extraction and quality assessment of each article was carried out independently by SRV and SK. Quality assessment and risk of bias was assessed using a standardized form consisting of 22 criteria (Additional file 1) [7], including information about study setting, population and design, sample selection, baseline characteristics, losses to follow-up, reliability of exposure and outcome measurements, reporting, the appropriateness of data analyses, confounding factors adjusted for and the study results. Discrepancies between assessors were resolved by discussion. Scores indicated a low (>16), medium (12-16) or high (<12) risk of bias.

## Results

Of 38 articles (34 observational studies and four double blind RCTs) included for review there were 12 for BMI or gestational weight gain; three for vitamin D, 14 for folate (of which six also examined B12 and another examined iron), seven for vitamin B12, eight for iron and one for dietary carbohydrate/protein intake. Although the mean age of the children was 19 years in the only study that we found for carbohydrate/protein intake, the age range of the participants (16-22 years) overlapped with the target age and hence we included the study in our review. There were no studies on vitamins B1 and B6.

Thirty four of the 38 articles were published in the last 10 years. Ages at which children were assessed ranged from 1 month to 17 years. Almost all studies adjusted for a range of potential confounders, and these are detailed in the relevant tables (Tables 2, 3, 4, 5, 6 and 7).

**Table 2:** Summary of the studies examining associations of maternal anthropometry with offspring cognitive function

Author, Year, Sample size, Age, Country, Study design	Maternal anthropometry	Cognitive function	Results after adjustment for confounders	QS and RB
^8^Neggers YH; 2003 * N* = 355 Age 5.3 years USA Prospective Low income African- Americans; mothers participated in Zinc supplementation trial	Pre-pregnancy BMI (kg/m^2^) BMI 4 categories Underweight (BMI < 19.8): 6.5 % Normal (19.8-26.0): 39.2 Overweight (26.1-29.0): 14.4 % Obese(>29): 39.9 % Gestational weight gain (kg)	Differential Ability Scale –general IQ (intelligence quotient), verbal and non-verbal abilities Peabody Gross Motor Scales	↑Pre-pregnancy BMI -↓ general IQ (β = -0.25) and non-verbal score (β = 0.29) Compared to children of normal weight mothers, children of obese mothers scored lower in general IQ (β = -4.7) and non-verbal abilities (β = -5.6) but not in verbal or motor skills Compared to children of normal weight mothers, children of underweight mothers scored lower in general IQ, verbal and non-verbal abilities but not significant No association between pregnancy weight gain and cognitive or motor skills Confounders adjusted for: Child’s BWT, GA, current age, MA, MS, maternal alcohol intake, MIQ, HE, child care status, zinc supplementation status	14 Medium
^9^Heikura U; 2008 Two birth cohorts 1966 * N* = 12058 Age 11.5 years Finland 1986 birth cohort * N* = 9432 Age 11.5 years Finland	Pre-pregnancy BMI (kg/m^2^) BMI 4 Categories 1966 cohort Thin (BMI < 20): 13.4 % Normal (BMI 20-24.9): 65 % Overweight (BMI 25.0–29.9): 17.9 % Obese (BMI ≥30): 3.8 % 1986 cohort Thin (BMI < 20): 24.3 % Normal (BMI 20-24.9): 58.7 % Overweight (BMI 25.0–29.9): 13.1 % Obese (BMI ≥30): 3.8 %	IQ (Standardised psychometric test or clinical developmental assessment): Test battery used not reported Intellectual disability (ID)-IQ < 70 severe ID (IQ < 50) mild (IQ 50-70)	Maternal pre-pregnancy obesity predicted ID in 1986 cohort (OR = 2.8) but not in 1966 birth cohort Low BMI associated with mild ID in 1966 cohort (OR = 2.1) Interaction between parity*BMI in 1966 cohort Higher risk of ID (OR = 2.9) in children of multiparous mothers with low BMI in 1986 cohort Confounders adjusted for: MA, SES (occupation), parity, place of residence, marital status	15 Medium
^10^Tanda R; 2012 * N* = 3412 Age 5-7 years USA Longitudinal	Pre-pregnancy BMI (kg/m^2^) and gestational weight gain (kg) BMI 4 categories Underweight (BMI < 18.5): 7.2 % Normal (BMI 18.5-24.9): 65.6 % Overweight (BMI 25.0-29.9): 17.6 % Obese (BMI ≥30): 9.6 %	Peabody Individual Achievement Test Reading and Mathematics scores	Pre-pregnancy obesity, but not overweight, was negatively associated with cognitive skills Compared to children of normal weight mothers, children of obese mothers scored 3 points lower (0.23 SD) in reading and 2 points lower (0.16 SD) in mathematics score ↑gestational weight gain - ↓ cognitive skills but not significant Confounders adjusted for: the child’s sex, GA, current age and body size, ethnicity, parity, SES (income), MA, ME, MIQ, HE	15 Medium
^11^Hinkle SN; 2012 * N* = 6850 Age 2 years USA Population based Longitudinal-Birth cohort	Pre-pregnancy BMI (kg/m^2^) and Gestational weight gain (kg) BMI 5 categories Underweight (BMI < 18.5): 5 % Normal (BMI 18.5-24.9): 56 % Overweight (BMI 25.0-29.9): 25 % Obese1(BMI 30.0-34.9): 8 %. Obese2 and 3(BMI > =35.0-39.9): 6 %	Bayley Scales of Infant Development –II (Mental Development Index (MDI) and Psychomotor Development Index (PDI))	Compared to the children of normal BMI mothers, children of mothers in all the other categories scored lower MDI, but significant in obese2 and 3 categories (β = 2.13 points) Risk of delayed mental development (<-1SD v > 1SD) observed in children of mothers with underweight (RR = 1.36) and extreme obese (RR = 1.38) categories No association between pre-pregnancy BMI and PDI Confounders adjusted for: the child’s sex, BWT, GA, BF, MA, ethnicity, marital status, parity, DM, PIH, ME, MS, SES (income)	16 Medium
^12^Basatemur E; 2012 Age 5 years (*n* = 11025) Age 7 years (*n* = 9882) UK Prospective population based birth cohort	Pre-pregnancy BMI (kg/m^2^) BMI continuous and categories BMI 4 categories Underweight (BMI < 18.5): 5.3 % Normal (BMI 18.5-24.9): 65.6 % Overweight (BMI 25.0-29.9): 20.1 % Obese (BMI ≥30): 9 % Excluded BMI < 16	5 Y-British ability scales-II Expressive language, nonverbal reasoning and spatial visualization 7 Y- British ability scales-II spatial visualization, verbal ability, and number skills test (National foundation for educational research progress in Math tests)	Children of underweight, overweight and obese mothers scored lower mean scores (0.1-0.3 SD) Maternal pre-pregnancy BMI is negatively associated with children’s general cognitive ability at 5 years (β = -0.075) and 7 years (β = -0.17) 5 years - ↑maternal BMI -↓Spatial visualization but no association with expressive language and nonverbal reasoning 7 years- ↑maternal BMI -↓Spatial visualization, verbal ability and number skills Confounders adjusted for: The child’s sex, current age, BWT, BMI, ethnicity, MA, ME, PE, SES, income, MS, DM	15 Medium
^13^Buss C; 2012 * N* = 174 Age 7.3 years USA Population based prospective Longitudinal-Birth cohort	Pre-pregnancy BMI (kg/m^2^) and Gestational weight gain (kg) BMI continuous and categories BMI 3 categories Normal (BMI 18.5-24.9): 58 % Overweight (BMI 25.0-29.9): 25.9 % Obese (BMI ≥30): 16.1 % Excluded underweight mothers	Executive function Continuous Performance Task (Go/No go task)	Higher pre-pregnancy BMI (continuous and categorical) was associated with impaired performance on the Go/No go task (F_1.157_ = 8.37 and F_2.156_ = 3.57 respectively) Children of obese mothers scored higher in performance measure (higher score indicates poor performance) compared to children of normal weight mothers. No difference in scores of performance efficiency between children of obese mothers vs children of overweight/normal weight mothers (Chen’s *d* effect size 0.62 SD) Gestational weight gain was not associated with child performance on the Go/No go task (F_1.157_ = 0.27) Confounders adjusted for: The child’s sex, current age, BMI, ethnicity, GA, parity, BWT, ME, MIQ, depression, obstetric risk (PIH, DM)	13 Medium
^14^Brion M; 2011 ALSPAC: population based prospective cohort UK * N* = ~5000 Age 38 months; Age 8 years Generation R: Population based pregnancy cohort Netherlands. * N* = ~2500 Age 30 Months	Pre-pregnancy BMI (kg/m^2^) Underweight (BMI < 18.5) Normal (BMI 18.5-24.9) Overweight (BMI 25.0-29.9) Obese (BMI ≥30) ALSPAC Normal BMI: 78.7 % Overweight/obese: 21.3 % Generation R Normal BMI: 77.9 % Overweight/obese: 22.1 % Excluded obese group (cognitive assessment at age 30-38 months)	ALSPAC-Verbal skills-MacArthur Toddler Communication Questionnaire maternal report Non-verbal skills-Diagnostic Analysis of Non-verbal Accuracy Test General intelligence-Wechsler Intelligence Scale for Children-II at 8-years Generation-R-Verbal skills-Dutch translation of the Language Development Survey Non-verbal-Dutch version of parent report of children’s abilities	ALSPAC: No association of maternal overweight with verbal and non-verbal skills. Maternal obesity was associated with ↓IQ (OR = 0.84) at 8 years Generation-R: no association between maternal overweight with verbal and non-verbal skills. Confounders adjusted for: ME, PE, occupation, income, social class (ALSPAC only), MS, BF	15 Medium
^15^Casas M; 2013 INMA: population based prospective birth cohort Spain * N* = ~1967 Age 11-22 months RHEA: Population based prospective cohort Greece * N* = 412 Age 17-20 months	Pre-pregnancy BMI (kg/m^2^) Underweight (BMI < 18.5) Normal (BMI 18.5-24.9) Overweight (BMI 25.0-29.9) Obese (BMI ≥30) INMA: 72.9 %, 19.2 % and 8 % normal, overweight and obese respectively RHEA: 68.3 %, 20.1 %, and 11 % normal, overweight and obese respectively Excluded underweight	INMA: Bayley Scales of Infant Development –I (Mental and Psychomotor scale) RHEA: Bayley Scales of Infant Development –III (Cognitive and fine and gross motor development scale)	Pre-pregnancy obesity, but not overweight, was negatively associated with cognitive skills Compared to children of normal weight mothers, children of obese mothers scored 2.67 points lower (INMA) and 3.57 points lower (RHEA and not significant) in mental (INMA) and cognitive development (RHEA) Cognitive score ↓ with increasing BMI (INMA -0.17 per kg/m^2^; RHEA -0.26 per kg/m^2^(not significant) No association of overweight/obesity with motor development in both cohorts Confounders adjusted for: gender, parental education, age, social class (only in INMA), maternal country of birth, breast-feeding duration, MS, employment status during pregnancy and after birth, parity, nursery attendance and main child minder	14 Medium
^16^Craig WY; 2013 Study 1- USA Population based cohort * N* = 101 Age 2 years Study 2- USA Population based cohort * N* = 118 Age 8 years In both studies participants were from control group of a case–control study	Pregnancy BMI (kg/m^2^; 2nd trimester) Normal (BMI 18.5-24.9) Overweight (BMI 25.0-29.9) Obese (BMI ≥30) Study 1: 31.6 %, 38.6 % and 29.7 % normal, overweight and obese respectively Study 2: 64.4 %, 25.4 %, and 10.2 % normal, overweight and obese respectively No underweight category	Study 1- Bayley Scales of Infant Development –III Cognitive, language and motor (gross and fine) domains Study 2-Wechsler Intelligence Scale for Children (WISC)-III Full-scale IQ, verbal and performance IQ	Study 1: ↑BMI categories- ↓scores for cognitive, language and motor domains (not significant) Percentage of children with ≥ 1 score below BSID-III score of 85 increased with BMI category and was higher among children of obese mothers compared to children of normal BMI mothers (OR 3.9) Study 2: ↑BMI categories- ↓scores for performance IQ but not for full-scale and verbal IQ Percentage of children with ≥ 1 score below WISC-III score of 85 increased with BMI category and was higher among children of obese mothers compared to children of normal BMI mothers (OR 5.2) Confounders adjusted for: gender, maternal age, smoking, number of prior births, SES (based on occupation and education)	14 Medium
^17^Huang L; 2014 * N* = 30212 Age 7 years USA Population based prospective cohort	Pre-pregnancy BMI (kg/m^2^) Gestational weight gain (lb) Underweight (BMI < 18.5)-9.1 % Normal (BMI 18.5-24.9)-69.2 % Overweight (BMI 25.0-29.9)-16.0 % Obese (BMI ≥30)-5.7 %	Wechsler Intelligence Scale for Children-I Full-scale, verbal and performance IQ	Pre-pregnancy obesity, but not overweight, was negatively associated with offspring IQ Compared to children of normal mothers, children of obese mothers scored 2 points lower in full-scale IQ and 2.5 points lower verbal IQ; compared to normal mothers children of underweight mothers scored ~0.6 points lower in full-scale IQ Suboptimal, inadequate or excessive weight gain in normal mothers-↓ full-scale IQ scores (~1-3 points) in children Excessive, but not inadequate, weight gain in obese mothers-↓ IQ scores in children Compared to children of normal mothers who gained 21-25 lb, children of obese mothers who gained >40 lb scored 6.5 points lower in full-scale IQ scores Confounders adjusted for: Maternal race, parity, MA, ME, MS, SES, marital status	15 Medium
^18^Tavris DR; 1982 * N* = 2789 Age 5 years USA Prospective longitudinal	Maternal gestational weight gain (difference in weight between first and last prenatal visits) 3 categories of weight gain 1) -24 to 4 lb, 2) 5 to 29 lb 3) ≥30 lb	Raven’s Coloured Progressive Matrices Details of cognitive domains assessed not mentioned	Children of mothers who gained <5 lb and >30 lb scored poorly compared to 2nd category (F = 3.23) Compared to 1st and 2nd category – no difference. Compared to 2nd and 3rd category- second category scored better (F = 4.31) Confounders adjusted for: Ethnicity, MA, parity, pre-pregnancy weight/height ratio, GA, ME, PE, income	13 Medium
^19^Gage SH; 2013 ALSPAC: population based prospective cohort-UK. * N* = 5832: Age 4 years * N* = 5191; Age 8 years * N* = 7339; Age 16 years	Maternal gestational weight gain (kg) 3 categories of weight gain 1: less than recommended 2: As recommended 3: more than recommended Pre-pregnancy weight (kg)	School Entry Assessment Score-4 years IQ- Wechsler Intelligence Scale for Children -III-8 years Adequate final exam results-16 years	Children of women gained weight < expected-↓ school entry assessment score (-0.075 SD) and adequate final-exam results (OR = 0.88); ↑Weight gain - early and mid pregnancy -↑school entry assessment score (0.072 and 0.077 SD) ↑ Weight gain in all three periods of pregnancy-↑ IQ at 8 years (0.070 to 0.078 SD) and ↑Pre-pregnancy weight-↓ school entry assessment score (-0.004 SD/kg), IQ (-0.004 SD) and the odds (OR = 0.99) of achieving adequate final exam results Confounders adjusted for: the child’s sex, current age, MA; ME, parity, pre-pregnancy BMI, smoking and mode of delivery	16 Medium

*BMI* body mass index, *QS* quality score, *RB* risk of bias, *GA* gestational age, *MA* maternal age, *BWT* birthweight, *SES* socio-economic status, *ME* maternal education, *PE* paternal education, *MIQ* maternal intelligence, *HE* home environment, *MS* maternal smoking, *BF* breast-feeding, *DM* maternal diabetes, *PIH* pregnancy induced hypertension

**Table 3:** Summary of the studies examining associations of maternal vitamin D status with offspring cognitive function

Author, Year, Sample size, Age, Country, Study design	Nutrient	Cognitive function	Results after adjustment for confounders	QS and RB
^20^Gale C; 2008 * N* = 178 Age 9 years UK Prospective longitudinal	Serum vitamin D concentrations assessed at 28-42 weeks gestation 21.2 % had <27.5 nmol/L 28.3 % had 27.5-50 nmol/L	Wechsler Abbreviated Scale of Intelligence Full-scale, verbal or performance IQ	No association between vitamin D concentrations and offspring IQ (full-scale, verbal or performance) Confounders adjusted for: Unadjusted	16 Medium
^21^Whitehouse AJO; 2012 Age 5 years (*n* = 534) Age 10 years (*n* = 474) Australia Prospective longitudinal	Serum vitamin D concentrations assessed at 18 weeks gestation 25.2 % mothers had insufficiency (≤46 nmol/L) (lowest quartile)	Peabody Picture Vocabulary Test Receptive language	Children of mothers with vitamin D insufficiency (lowest quartile (≤46 nmol/L) were at increased risk (OR = 1.97) of language impairment compared to children of mothers without insufficiency (highest quartile (≥72 nmol/L; OR = 1.00) Confounders adjusted for: MA, MS, parity, family income, season of maternal blood sampling	13 Medium
^22^Morales E; 2012 * N* = 1820 Age 11-23 Months Spain Prospective population based cohort study	Plasma vitamin D concentrations assessed during 12-23 weeks gestation 19.5 % mothers had deficiency (<20 ng/ml) 31.5 % had insufficiency (20-30 ng/ml)	Bayley Scales of Infant Development (mental (MDI) and psychomotor (PDI) developmental score)	A positive linear association between vitamin D concentrations and MDI and PDI Per 10 ng/ml increase in vitamin D concentrations MDI and PDI score increased by β = 0.79 and β = 0.88 points respectively Compared to infants of deficient mothers, infants of mothers with normal level scored higher MDI (β = 2.60) and PDI (β = 2.32) points respectively Confounders adjusted for: The child’s sex, BWT, area of study, maternal country of origin, MA, parity, pre-pregnancy BMI, SES, ME, MS, alcohol and season	15 Medium

*QS* quality score, *RB* risk of bias, *MA* maternal age, *BWT* birthweight, *ME* maternal education, *MS* maternal smoking, *BMI* body mass index, *SES* socio-economic status

**Table 4:** Summary of the studies examining associations of maternal folate status with offspring cognitive function

Author, Year, Sample size, Age, Country, Study design	Nutrient	Cognitive function	Results after adjustment for confounders	QS and RB
^23^WU BTF; 2012 * N* = 154 Age 18 Months Canada Prospective	Plasma folate and tHcy concentrations assessed at 16 and 36 weeks gestation No folate deficiency (plasma folate <6.8 nmol/l) High tHcy not reported	Bayley Scales of Infant Development Receptive language, expressive language, cognitive skills, fine motor and gross motor	No association of folate and tHcy with cognitive function Confounders adjusted for: The child’s sex, BF, ethnicity, MA, MIQ, maternal fatty acid level	13 Medium
^24^Tamura T; 2005 * N* = 355 Age 5 years USA Prospective Mothers participated in zinc supplementation trial during pregnancy	Red cell and plasma folate concentrations – 19, 26 and 37 weeks gestation and tHcy concentrations-26 and 37 weeks Low folate-(plasma folate <11 nmol/L) 19 weeks- 7.4 %; 26 weeks- 8.2 %; 37 weeks- 14.0 % Red cell folate <430 nmol/L) 19 weeks- 7.2 %; 26 weeks- 3.8 %; 37 weeks- 3.3 % High tHcy (tHcy > 7 μmol/L) 26 weeks- 8.4 %; 37 weeks- 22.1 %	Differential Ability Scale (verbal, nonverbal and General IQ), Visual and Auditory Sequential Memory (visual and auditory memory span) Knox Cube (attention span and short-term memory) Gross Motor Scale (Gross motor development and Grooved Pegboard (manipulative dexterity)	No difference in the mental and psychomotor developmental scores between children of mothers with normal and deficient folate and tHcy groups. No difference in test scores even across range of folate status (quartiles) Confounders adjusted for: The child’s sex, GA, BWT, MA, BMI, MS, MIQ, alcohol and drug use, HE	15 Medium
^25^Bhate V; 2008 * N* = 108 Age 9 years India Prospective community based birth cohort	Erythrocyte folate and tHcy concentrations assessed at 28 weeks gestation No details about low folate or high tHcy concentrations	Raven’s Coloured Progressive Matrices-Intelligence; Visual recognition Colour Trial Test-sustained attention and executive function Digit-span test-short-term or working memory	No association of erythrocyte folate, tHcy with any of the cognitive tests Confounders adjusted for: The child’s sex, age, education, weight and head circumference, B12 level, SES, education of the head of the family	14 Medium
^26^Veena SR; 2010 * N* = 536 Age 9-10 years India Prospective birth cohort	Plasma folate and tHcy concentrations assessed at 30 ± 2 weeks gestation Low folate-(folate <11 nmol/L)-4 % High tHcy (tHcy > 7 μmol/L)-3 %	Kauffman Assessment Battery for Children-II Learning, long-term retrieval, short-term memory and reasoning Wechsler Intelligence Scale for Children-III attention and concentration Koh’s block design visuo-spatial ability Verbal fluency	No difference in all the cognitive test scores between folate deficient and normal groups. ↑folate concentrations (SD)-↑learning (0.10 SD), visuo-spatial ability (0.10 SD) and attention and concentration (0.10 SD) No association between tHcy concentrations across the entire range or hyperhomocysteinemia and cognitive function Confounders adjusted for: The child’s sex, GA, age, education, weight and head circumference at birth, parity, MA, maternal BMI, ME, PE, SES, religion, rural/urban residence, the child’s current head circumference, BMI and folate concentrations	16 Medium
^27^Gross RL; 1974 * N* = 32 Age 6 weeks to 4 years Africa Case–control study	Folic acid deficiency (based on bone marrow exam or serum folate level) (Hb 3.2-8.9 g %)	Denver Developmental Screening Test (gross motor, fine motor, language and personal-social)	Folic acid deficiency was associated with abnormal or delayed development on one or more of the 4 areas examined Confounders adjusted for: No information	6 High
^28^Del Rio Garcia; 2009 * N* = 253 Age Infancy (1- 12 months) Mexico Prospective birth cohort	Daily dietary intake of folate (first trimester FFQ) Deficient daily folate intake (<400 μg) -70 %	Bayley Scales of Infant Development -II (Mental Development Index(MDI) and Psychomotor Development Index (PDI))	Folate intake deficiency-↓ MDI (β = -1.8) in infants of mothers who were carriers of MTHFR677 TT genotype Confounders adjusted for: BWT, BF, current age, energy intake at age 6 months, maternal BMI, pregnancy hypertension, ME, HE and MTHFR 1298A > C genotype	16 Medium
^29^Villamor E; 2012 * N* = 1210 Age 3 years USA Prospective pre-birth cohort	Average daily intake of folate at 1st and 2nd trimester (FFQ + Supplements) Peri-conceptional intake of folate from supplements (LMP-4 weeks gestation)	Peabody Picture Vocabulary Test-Receptive Language Wide Range Assessment of Visual Motor Abilities-visual-motor; visual-spatial and fine motor	First but not 2nd trimester folate intake (food + supplement) positively related to receptive language but not with visuo-motor abilities. Every increment of 600 μg/day folate intake -↑1.6 points receptive language. No association of peri-conceptional folate intake with cognitive function Confounders adjusted for: MA, parity, ethnicity, MS, pre-pregnancy BMI, ME, PE, MIQ, energy, fish and iron intake, income, the child’s sex and English as primary language	15 Medium
^30^Boeke C; 2013 * N* = 895 Age 7 years USA Prospective pre-birth cohort	Average daily intake of folate at 1st and 2nd trimester (FFQ + Supplements)	Peabody Picture Vocabulary Test-Receptive Language Wide Range Assessment of Memory and Learning-II edition, Design and Picture Memory subtests: visuo-spatial memory Kaufman Brief Intelligence Test-II edition Verbal and non-verbal intelligence	No association of folate intake with cognitive function Confounders adjusted for: MA, parity, ethnicity, MS, pre-pregnancy BMI, ME, PE, MIQ, energy, fish and iron intake, income, the child’s sex and English as primary language	16 Medium
^31^Wehby GL; 2008 * N* = 6774 Age 3 years USA Population based longitudinal	Folic acid supplements (3 months prior to pregnancy and/or during the following 3 months) 3 % used supplement	Denver developmental screening-language, personal-social, gross motor and fine motor	Folic acid use was associated with improved gross motor development (OR = 0.5) Confounders adjusted for: The child’s sex, age, ethnicity, MA, ME, MS, alcohol, drug abuse, income, maternal health status	11 High
^32^Roth C; 2011 * N* = 38954 Age 3 years Prospective observational Norway	Folic acid supplements with or without other supplements (4 wks before to 8 wks after conception) 18.9 % used only folic acid 50 % used folic acid + other supplements	Language Grammar Rating scale - Language delay (severe and moderate) Severe-children with minimal expressive language i.e. only 1 word or unintelligible utterances; Moderate-children can produce 2-3 word phrases Gross motor skills-Ages and Stages questionnaire	Use of folic acid resulted in reduced risk of severe (OR = 0.55) and moderate language delay (OR = 0.80) No association between folic acid intake and delay in gross motor skills Confounders adjusted for: Maternal marital status, BMI, parity and education	17 Low
^33^Forns J; 2012 * N* = 393 Age 11 years Population based prospective birth cohort; Spain	Folic acid supplements with or without other vitamins Dose and duration: No information. 66.8 % used folic acid + other supplements	Continuous Performance Test (Attention function) Omission error; Commission error HRT-mean response time (for correct hits)	Supplementation with folic acid reduced the incidence rate ratio (IRR = 0.80) of omission errors No association with commission and HRT Confounders adjusted for: Parity, PE, social class, MIQ, maternal mental health, MS, BWT, BF	14 Medium
^34^Julvez J; 2009 * N* = 420 Age 4 years Population based prospective birth cohort Spain	Folic acid supplements with or without other vitamins Dose and duration: No information 34 % used only folic acid 24 % used folic acid + other supplements	McCarthy Scales of Children’s Abilities General cognitive scale and subscales (Verbal, perceptive-performance, memory, quantitative and motor) and executive function (Verbal and perceptive-performance)	Use of maternal folic acid supplement was positively associated with verbal (general cognitive) score (β = 3.98) and verbal (executive function (β = 3.97)), motor skills (β = 4.55) Confounders adjusted for: The child’s sex, age, school season, area of residence, GA, BF, parity, maternal marital status, MS, use of calcium and iron supplements, ME, PE and social class	14 Medium
^35^Holmes-Siedle; 1992 * N* = 96 Age 2-5 years UK Prospective observational	Peri-conceptional multivitamin containing folic acid (0.36 mg) supplements daily with other vitamins and minerals (Minimum 28 days before conception until the second missed menstrual period)	Denver developmental screening test (DDST) (language, motor and social skills)	No significant difference in development score among supplemented group compared to general population Confounders adjusted for: No information	12 Medium
^36^Campoy C; 2011 * N* = 154 Age 6.5 years Double blind randomized controlled trial European centres (Germany, Spain and Hungary)	4 supplement (milk based) groups Fish oil (*N* = 37) 5-methyl tetrahydrofolate-400 μg (*N* = 37) Fish oil + 5-methyl tetrahydrofolate (*N* = 35) Placebo (*N* = 45) Daily supplementation from 20th week of gestation until delivery Plasma/erythrocyte folate concentrations during 2nd and 3rd trimester and at the time of delivery; No information about compliance	Kaufman Assessment Battery for Children (KABC): Sequential processing scale Simultaneous processing scale Mental Processing Composite (MPC)	No significant difference in cognitive scores between supplement groups No association of maternal plasma or erythrocyte folate concentrations during pregnancy and at the time of delivery with cognitive function Confounders adjusted for: Unadjusted	19 Low

*QS* quality score, *RB* risk of bias, *tHcy* total homocysteine, *Hb* haemoglobin, *LMP* last menstrual period, *FFQ* food frequency questionnaire, *BMI* body mass index, *GA* gestational age, *MA* maternal age, *BWT* birthweight, *BF* breast-feeding, *SES* socio-economic status, *ME* maternal education, *PE* paternal education, *MIQ* maternal intelligence, *HE* home environment, *MS* maternal smoking, *MTHFR* methylenetetrahydrofolate reductase

**Table 5:** Summary of the studies examining associations of maternal vitamin B12 status with offspring cognitive function

Author, Year, Sample size, Age, Country, Study design	Nutrient	Cognitive function	Results after adjustment for confounders	QS and RB
^23^WU BTF; 2012 * N* = 154 Age 18 Months Canada Prospective	Plasma vitamin B12 and holotranscobalamin concentrations assessed at 16 and 36 weeks gestation 7.8 % low B12 (<148 pmol/l)	Bayley Scales of Infant Development Receptive language, expressive language, cognitive skills, fine motor and gross motor	No association of B12 and holotranscobalamin with cognitive function Confounders adjusted for: The child’s sex, BF, ethnicity, MA, MIQ, maternal fatty acid level	13 Medium
^25^Bhate V; 2008 * N* = 108 Age 9 years India Prospective community based	Plasma Vitamin B12 concentrations assessed at 28 weeks gestation B12 status 2 groups Lowest <77 pmol/L Highest >224 pmol/L	Raven’s Coloured Progressive Matrices-Intelligence Visual recognition Colour Trial Test-sustained attention and executive function Digit-span test-short-term or working memory	Children in group 1 performed slowly in sustained attention (182 seconds Vs 159) and short-term memory (2.6 digits Vs 2.9) No association with other tests Confounders adjusted for: The child’s sex, age, education, weight and head circumference, B12 level, SES, education of the head of the family	14 Medium
^26^Veena SR; 2010 * N* = 536 Age 9-10 years India Prospective birth cohort	Plasma vitamin-B12 concentrations assessed at 30 ± 2 weeks gestation Low B12-(B12 < 150 pmol/L)-42 %	Kauffman Assessment Battery for Children-II-Learning, long-term retrieval, short-term memory and reasoning Wechsler Intelligence Scale for Children-III-attention and concentration Koh’s block design-visuo-spatial ability Verbal fluency	No association between B12 concentrations and cognitive function No difference in mean score between children of mothers with low and normal B12 status Confounders adjusted for: The child’s sex, GA, weight and head circumference at birth, parity, MA, maternal BMI, ME, PE, SES, religion, rural/urban residence, the child’s current age, education, head circumference, BMI and B12 concentrations	16 Medium
^28^Del Rio Garcia; 2009 * N* = 253 Age Infancy (1- 12 months) Mexico Prospective birth cohort	Daily dietary intake of vitamin B12 (first trimester FFQ) Deficient daily dietary intake (B 12 < 2.0 μg/day) – 21.3 %	Bayley Scales of Infant Development -II (Mental Development Index(MDI) and Psychomotor Development Index (PDI))	B12 intake deficiency-↓mental development (β = -1.6 points) Confounders adjusted for: BWT, BF, current age, energy intake at age 6 months, maternal BMI, pregnancy hypertension, ME, HE and MTHFR 1298A > C genotype	16 Medium
^29^Villamor E; 2012 * N* = 1210 Age 3 years USA Prospective pre-birth cohort	Average daily intake of vitamin B12 - 1st and 2nd trimester (FFQ + Supplements) Peri-conceptional B12 intake from supplements (LMP - 4Wks gestation)	Peabody Picture Vocabulary Test-Receptive Language Wide Range Assessment of Visual Motor Abilities-visual-motor; visual-spatial and fine motor	↑ B12 intake (2.6 μg/day) during 2nd trimester (not 1st trimester) -↓ (0.4 points) receptive language No association of peri-conceptional B12 intake with cognitive function Confounders adjusted for: MA, parity, ethnicity, MS, pre-pregnancy BMI, ME, PE, MIQ, energy, fish and iron intake, income, the child’s sex and English as primary language	15 Medium
^30^Boeke C; 2013 * N* = 895 Age 7 years USA Prospective pre-birth cohort	Average daily intake of B12 at 1st and 2nd trimester (FFQ + Supplements)	Peabody Picture Vocabulary Test-Receptive Language Wide Range Assessment of Memory and Learning-II edition, Design and Picture Memory subtests: visuo-spatial memory Kaufman Brief Intelligence Test-II edition Verbal and non-verbal intelligence	No association of B12 intake with cognitive function Confounders adjusted for: MA, parity, ethnicity, MS, ME, PE, MIQ, HE, intake of energy, fish and other methyl donors, the child’s sex and current age	16 Medium
^37^Bonilla C; 2012 * N* = 6259 Age 8 years UK Population based prospective birth cohort	Daily dietary vitamin B12 intake. (FFQ; 3rd trimester-32 weeks)	Wechsler Intelligence Scale for Children-III- Full-scale IQ	No association between maternal B12 intake and child’s IQ. Confounders adjusted for: The child’s sex, GA, BWT, BF, current age, MA, parity, ME, social class, MS, alcohol, maternal energy intake and infections in pregnancy, folate supplementation	14 Medium

*QS quality score*, *RB* risk of bias, *LMP* last menstrual period, *BMI* body mass index, *FFQ* food frequency questionnaire, *BWT* birthweight, *SES* socio-economic status, *GA* gestational age, *MA* maternal age, *ME* maternal education, *PE* paternal education, *MIQ* maternal intelligence, *HE* home environment, *MS* maternal smoking, *BF* breast-feeding, *MTHFR* methylenetetrahydrofolate reductase

**Table 6:** Summary of the studies examining associations of maternal iron status with offspring cognitive function

Author, Year, Sample size, Age, Country, Study design	Nutrient	Cognitive function	Results after adjustment for confounders	QS and RB
^31^Wehby GL; 2008 * N* = 6774 Age 3 years USA Population based longitudinal	Prenatal iron supplements (3 months prior to pregnancy and/or during the following 3 months) 36.2 % used supplement	Denver developmental screening-language, personal-social, gross motor and fine motor	Iron use was associated with improved performance in personal-social development (OR = 0.5) but not with language and motor domains Confounders adjusted for: The child’s sex, age, ethnicity, MA, ME, MS, alcohol, drug abuse, income, maternal health status	11 High
^38^Rioux FM; 2011 * N* = 63 Age 6 Months Canada Observational	Hb, serum ferritin at 28-32 weeks gestation 90 % mothers took iron supplements (27 mg of iron)	Brunet-Lezine Scale of Psychomotor Development of Early Childhood. Bayley Scales of Infant Development	No association between maternal gestational Iron status with mental and psychomotor development. Confounders adjusted for: ME, PE, MIQ, income, BF, GA, BWT, birth head circumference, infants’ current weight and Hb	16 Medium
^39^Ferarouei. M; 2010 * N* = 9983 14 years * N* = 10474 16 years Finland Prospective Birth cohort study	Hb concentrations at 3rd 7th and 9th gestational months Anaemia	School performance 14 years- Self report 16 years- School report	↑maternal HB at 9 months-↑ total school performance score (β = 0.03) and theory score at 14 years and total score at 16 years Offspring of mothers with anaemia –low school scores (OR = -0.05 at 14 years and (OR = -0.06) at 16 years Confounders adjusted for: The child’s sex, BWT, pregnancy wanted or not, ME, social class, parity, marital status, MS, maternal mental health status	18 Low
^40^Davidson PW; 2008 * N* = 229 Age 5, 9, 25and 30 months Republic of Seychelles Longitudinal cohort study	Iron- total body stores at 14-24 weeks of gestation assessed before the start of iron supplementation	Bayley Scales of Infant Development: Mental Development Index (MDI) and Psychomotor Development Index (PDI) (9 and 30 months) Infant cognition (Fagan Infantest-novelty preference) and Visual Expectation Paradigm –visual recognition memory (9 and 25 months) A-not-B and Delayed Spatial Alternation: inhibition, working memory, planning and attention (25 months)	No association between maternal iron stores and cognitive function at any age. Confounders adjusted for: The child’s sex, BWT, MA, SES, HE, MIQ and both parents living with the child (yes/no)	19 Low
^41^Lewis SJ; 2013 * N* = ~3,500 Age 8 years UK Population based prospective birth cohort	Hb concentrations Before 18 weeks Hb <11.0 g/dl -8 % After 28 weeks Hb <11.0 g/dl -30 %	Wechsler Intelligence Scale for Children-III- Full- scale IQ	No association between maternal Hb and child’s IQ Confounders adjusted for: GA, ME, the child’s genotype, iron supplementation, population stratification	15 Medium
^42^Tran TD; 2013 * N* = 378 Age 6 months Vietnam Population based prospective cohort study	Iron deficiency anaemia (Hb <11.0 g/dl and serum ferritin <15 ng/ml) during 12-28 weeks of gestation-16 %	Bayley Scales of Infant and Toddler Development-III edition-cognitive score	Infants of anaemic mothers scored 11.6 points (0.77SD) lower in BSID cognitive scores compared to infants of non-anaemic mothers. Confounders adjusted for: parity, MA, ME, wealth index, the child’s birth and current weight, family support, BF.	18 Low
^43^Zhou SJ; 2006 * N* = 302 Age 4 years; Australia Double blind randomized controlled trial	Iron supplements (20 mg/day) or placebo from 20 weeks gestation until delivery Compliance-86 %	Stanford Binet Intelligence Scale – IQ (verbal reasoning, visual reasoning, quantitative reasoning and short-term memory)	No difference between the children of supplement group and placebo group in the mean score of composite IQ or any subscales IQ or in the proportion of children whose IQ fell 1 or 2 SD below the mean. Confounders adjusted for: sex, birth order, gestational age, MA, ME, PE, HE, BF	20 Low
^44^Li Q; 2009 * N* = 1305 Age 3, 6 and 12 months Double blind cluster randomized controlled trial China	3 intervention groups (All received folic acid) Folic acid alone 400 μg (*n* = 471)-control Iron 60 mg + folic acid 400 μg (*n* = 438) Multiple micronutrients ((b vitamins (1,2,3 6 AND 12), vitamin A, D, C, E and minerals (zinc, iodine, copper, selenium) + Iron 30 mg + 400 μg folic acid)) (*n* = 396) Daily supplementation from enrolment until delivery; inadequate information about compliance	Bayley Scales of Infant Development :Mental (MD) and Psychomotor development (PD)	No significant difference in infants MD and PD score at 3 and 6 months and PD score at 12 months between supplement groups Mean MD score among children of multiple micronutrient group increased by 1 to 1.22 points compared to children of folic acid alone, or folic acid + iron group at 12 months Confounders adjusted for: Infants age, sex, gestational age, apgar score, BWT, infant health, maternal age and BMI, parental education, occupation, SES, number of tablets consumed	19 Low

*QS quality score*, *RB risk of bias*, *Hb haemoglobin*, *GA gestational age*, *MA maternal age*, *BWT birthweight*, *SES socio-economic status*, *ME maternal education*, *PE paternal education*, *MIQ maternal intelligence*, *HE home environment*, *MS maternal smoking*, *BF breast-feeding*, *BMI body mass index*

**Table 7:** Summary of the studies examining associations of maternal carbohydrate/protein with offspring cognitive function

Author, Year, Sample size, Age, Country, Study design	Nutrient	Cognitive function	Results after adjustment for confounders	QS and RB
^45^Alderman H; 2014 * N* = 6774 Age 16-22 years Double blind cluster randomized controlled trial Gambia	Carbohydrate/protein supplements Intervention Group: 2 biscuits daily (1015 kcal carbohydrate and 22 g protein) from 20 weeks of gestation to delivery Control group: same supplements for 20 weeks during postpartum but not during pregnancy No information about compliance	Raven’s progressive matrices- nonverbal reasoning ability The Mill Hill vocabulary test The backward and forward digit-span test Schooling achievement (questionnaire)	No difference in any of the cognitive test scores or schooling achievement between children whose mothers received the supplements during pregnancy and children whose mothers received supplements during postpartum. Confounders adjusted for: The child’s sex, age, GA, ME, PE, maternal height, parity, season of birth, language and village allocation	19 Low

*QS* quality score, *RB* risk of bias, *GA* gestational age, *ME* maternal education, *PE* paternal education

### Maternal anthropometry (Table 2)

All were observational studies and from developed countries [8–19]. Of the 12 studies, 10 examined pre-pregnancy BMI or weight, mostly self-reported [8–17], and six examined gestational weight gain [8, 10, 13, 17–19]. Sample size varied from 101 to over 30,000 mother-offspring pairs. Loss to follow-up was high (>30 %) in 7 studies [8, 10, 12, 14, 17–19]. Some studies excluded children of underweight mothers (BMI < 16 or <18.5 kg/m^2^) [12, 13, 15, 16].

In the 10 studies of pre-pregnancy BMI and/or weight exposure data were collected during antenatal visits [8–10, 13–19] or up to nine months following the index pregnancy or delivery [11, 12]. The Bayley Scale of Infant Development (BSID) was used in three studies [11, 15, 16] but different instruments were used, and different cognitive domains were assessed, in all the others. Assessment was by trained examiners in all except one, where it was based on parental report [14]. None of the papers stated whether outcome assessors were blind to exposure status.

The associations of maternal pre-pregnancy BMI and/or weight, or gestational weight gain with children’s cognitive function were mostly consistent. Nine of the ten studies using BMI categories found that maternal pre-pregnancy obesity (BMI > 29 kg/m^2^) was associated with lower offspring cognitive function than normal maternal BMI (Table 2) [8–13, 15–17]. The effect size was small. For example, in one study, mental development scores at age ~2 years were 0.1 SD lower in the extremely obese maternal BMI category (BMI > 35 kg/m^2^) [11]. Children of obese mothers scored ~0.3 SD lower in general cognitive and non-verbal abilities but not in verbal or motor abilities at age 5.3 years [8], ~0.1-0.2 SD lower in reading and mathematics scores at age 5-7 years [10] and in mental but not psychomotor development scores at age 1-2 years [15]. In another study, children of obese mothers scored ~2-2.5 points lower in full-scale intelligence quotient (IQ) and verbal-scale IQ but not in performance-scale IQ at age 7 years [17]. Similarly, in a study of two datasets (both with a small sample, *N* = ~100), in one dataset children of obese mothers scored 0.6 SD lower in performance IQ but not in full-scale and verbal IQ at age 8 years. In the other dataset children of obese mothers scored 0.2-0.5 SD lower in motor, language and cognitive scores at age 2 years, but this was not significant [16]. In this study, the percentage of children who scored below the composite scores in BSID at age 2 years and Wechsler Intelligent Scale for children (WISC-III) at age 8 years was higher in children of obese mothers (BSID: 33 % v 13 %; WISC-III: 50 % v 17 %) [16]. In another study that included two birth-cohorts, risk of intellectual disability (IQ score < 70) was higher in children of obese mothers in one cohort (OR = 2.8; 95 % CI: 1.5, 5.3) at age 11.5 years, but there was no association between maternal obesity and offspring intellectual disability in the other cohort [9]. In the remaining studies effects were found in both overweight and obese categories [12, 13]. Children of obese and overweight mothers scored ~0.1-0.2 SD lower in general intelligence at age 5 and 7 years [12] and performed poorly in a test of executive function at age 7 years [13]. There was no significant association between maternal overweight/obesity and the child’s cognitive function in only one of the 10 studies that included two cohorts [14].

Four of the 10 studies also reported an inverse association between maternal BMI (used as a continuous variable) and offspring cognitive function [8, 12, 13, 15]. For example, cognitive and psychomotor development scores fell by 0.01 SD per unit increase in maternal BMI [15].

In another study, for each kg increase in maternal pre-pregnancy weight there was a small reduction (0.004 SD) in offspring school entry assessment scores at age 4 years, IQ at age 8 years and odds of achieving adequate final exam results at age 16 years (OR = 0.99; 95 % CI: 0.98, 0.99) [19].

Six of the 10 studies also examined maternal underweight as a predictor of the children’s cognitive function. All were from developed countries and based on quite small numbers of underweight mothers. All six, however, found lower cognitive function among children of underweight (BMI <20 kg/m^2^) mothers compared to normal weight mothers [8–12, 17]. In one, there was a very small non-significant difference in general cognitive, verbal and non-verbal abilities scores (0.06-0.1 SD) at age 5.3 years [8] and reading and mathematics scores (0.02-0.05 SD) at age 5-7 years [10]. In a study that included two birth-cohorts, risk of mild (IQ score 50-70), but not severe intellectual disability (IQ score < 50), was higher in children of underweight compared to normal mothers in one cohort (OR = 2.1; 95 % CI: 1.0, 4.1) at age 11.5 years, but there was no association in the other cohort [9]. In another study, with no adjustment for confounders, there was a significantly lower general intelligence score (0.2-0.3 SD) at age 5 and 7 years [12]. In the fifth, there was a significantly higher risk of delayed mental development at age ~2 years (risk ratio 1.36; 95 % CI: 1.04, 1.78), but no association when the scores were treated as a continuous variable [11]. In the sixth, children of underweight mothers had lower full-scale IQ scores (~0.6 points; 95 % CI: -1.5, -0.1) [17].

Three out of the six studies that used gestational weight gain as the exposure found fairly consistent effects on offspring cognitive function. Less than expected weight gain in normal weight mothers or more than expected weight gain in normal or already obese mothers had a negative impact on offspring cognition. One found poorer non-verbal intelligence scores in children of mothers who gained >30 pounds (*N* = 230) compared to those who gained 5-29 pounds (*N* = 1361) [18]. In another study, children of mothers who gained less weight than recommended had lower school entry assessment scores (0.08 SD) at age 4 years and were less likely to achieve ‘adequate’ final exam results (OR = 0.88) at age 16 years [19]. There was a ~0.07 SD increase in offspring school entry assessment scores and a 0.07 SD increase in 8- year IQ, per 400 g/week gain in maternal weight during early, mid and late pregnancy. In a third study, the association of gestational weight gain with the child’s cognitive function differed in normal and obese mothers [17]. Either inadequate or excessive weight gain in mothers of normal weight was associated with lower offspring IQ scores at age 7 years [17]. Among obese mothers, inadequate gestational weight gain was unrelated to the children’s IQ, but higher gestational weight gain >40 pounds compared with 21-25 pounds was associated with lower full-scale IQ (-6.5 points; 95 % CI: -0.2, -0.11) [17]. The other three studies (two with a small sample (*N* = 355 [8]; *N* = 174 [13])) found no association between gestational weight gain and offspring cognitive function [8, 10, 13].

Risk of bias was medium in all 12 studies (score: 13-16). Factors most often responsible for a high risk of bias score were inadequate control for socio-economic status (SES), unmeasured confounders such as maternal intelligence (MIQ) and home environment, low validity of the exposure, small sample and questionable selection, poor reporting about losses to follow-up and observer bias.

### Maternal vitamin D (Table 3)

All three studies were conducted in developed populations and were observational [20–22]. Sample size varied from 178 to over 1800 mother-offspring pairs. The proportion of participants lost to follow-up was very high (70 %) in one study [20], high in another (30-40 %) [21] and small (12 %) in the third study [22].

All the studies assessed serum vitamin D concentration using stored samples collected during the second or third trimester. The duration of sample storage was 5 years in one study [20], 20+ years in another [21] and unreported in the third [22]. In all, cognitive function was assessed by trained personnel, though the cognitive domains and the test battery used were different. None reported whether the outcome assessors were blind to exposure status.

Of the three studies, one found a two-fold increase in language impairment in children of mothers with vitamin D deficiency (<46 nmol/L) compared to children of mothers with vitamin D >70 nmol/L [21]. Another study found higher mental and psychomotor development scores (2-3 score points (0.1-0.2 SD)) in children of mothers with normal vitamin D status (>75 nmol/L) compared to those with deficiency (<50 nmol/L) [22]. It also found a positive association between maternal vitamin D concentrations and offspring mental and psychomotor development scores (0.8-0.9 score points (~0.06 SD) per 25 nmol/L increase). The third study, with a small sample (*N* = 178) found no associations [20].

Risk of bias was medium in all three studies (score: 13-16).

### Maternal folate (Table 4)

Of 14 studies (13 observational [23–35] and one trial [36]), four (all observational) were conducted in developing countries [25–28]. The trial was in three Europeans centres (Germany, Spain and Hungary)) [36]. The sample size varied from 32 to over 39,000 mother-offspring pairs. Loss to follow-up was high (30-50 %) in four studies [24, 28, 30, 36], ~10-20 % in five [23, 26, 29, 33, 34], 1 % in one [35], and unreported in the others [25, 27, 31, 32].

The exposure was measured in different ways, and at different gestational ages. Four studies [23–26] used plasma/red cell folate and/or total homocysteine concentrations, assessed during the second and/or the third trimester. One African study used folic acid deficiency based on bone marrow or serum folate level [27]. Three studies used daily folate intake during the first and/or the second trimester (calculated from a food frequency questionnaire (FFQ) and/or supplement use) as the predictor [28–30]. One of these [29] and the remaining five studies used folic acid supplement use (with/without other vitamins and/or minerals) [31–35]. In two of them supplements were used 4 weeks-3 months prior to pregnancy and/or 2-3 months after conception [31, 32, 35]. In three others there were no details about initiation and duration of supplement use [29, 33, 34]. In the European trial pregnant mothers were supplemented daily with 400 μg of 5-Methyl-tetrahydrofolate alone, or fish oil with/without folate, or placebo from the 20th week of gestation until delivery [36].

Of the 14 studies, three used the Denver development scale [27, 31, 35], two used BSID [23, 28] and others used different batteries to assess cognitive function. The cognitive domains tested varied between studies but were assessed by trained investigators in all except two, which relied on parental report. [31, 32] Only two studies reported whether outcome assessors were blind to exposure status [24, 28].

The trial (*N* = 154, recruited in three European countries) found no difference in cognitive scores between the folic acid supplemented group and intervention group without folic acid at age 6.5 years [36].

Findings from the four observational studies that used plasma folate or homocysteine concentrations as the exposure were mostly consistently negative [23–26]. Three found no association of maternal folate or homocysteine concentrations with offspring mental and psychomotor development at age 1-2 years [23], verbal, non-verbal and general IQ, and gross motor development at age 5 years [24] and non-verbal intelligence, attention and memory at age 9 years [25]. One of them had a small sample (*N* = 154) of well-educated and affluent mothers who had no folate deficiency [23]. In another, mothers were from a socially disadvantaged background, without much variation in folate status [24]. In the third study the sample was very small (*N* = 108) and information about folate status was inadequate [25]. The other observational study, from India, found a positive association between third trimester maternal folate concentrations and the children’s learning, visuo-spatial ability and attention score (0.1 SD/SD increase in folate concentrations), but none with reasoning, short-term memory or verbal fluency scores, at age 9-10 years. There were no differences between children of deficient (4 %) or non-deficient mothers [26]. In this study there were no associations between maternal homocysteine concentrations and offspring cognitive function. In the African case–control study folate deficiency was associated with abnormal or delayed motor and/or language development at age 6 weeks-4 years [27]. This study had a very small sample size (*N* = 32) and a high risk of bias (score: 6).

In general findings were fairly consistently positive in the studies in which dietary folate intake was the exposure. In one study, low maternal folate intake (<400 μg/day) was associated with a lower mental development index (MDI, -1.8 score points (~0.3 SD); 95 % CI: -3.6, -0.04), but not psychomotor development index (PDI) score in children of mothers who were carriers of the Methylenetetrahydrofolate reductase (MTHFR) 677 TT genotype, but not in others, at age 1-12 months [28]. In another study, for each 600 μg/day increase in maternal folate intake from food and supplements during the first trimester, children’s receptive language scores increased by 1.6 score points (0.1 SD); 95 % CI: 0.1, 3.1 at age 3 years [29]. There were no associations with folate intakes peri-conceptionally, or in the second trimester, and no associations with children’s visuo-motor scores [29]. In another study, there were no associations of maternal folate intake at any stage of pregnancy with children’s cognitive function at age 7 years [30].

Four out of five observational studies of supplement use found positive associations between the use of folic acid supplements and offspring cognitive function [31–34]. In a study in the USA, supplement use was associated with better gross motor, but not fine motor or language development in the children at age 3 years (OR = 0.51; 95 % CI: 0.28, 0.93) and a lower risk of poor psychomotor development (OR = 0.48; 95 % CI: 0.25, 0.94) only in the sub-group of African-American children [31]. In another study, maternal supplement use was associated with a reduced risk of severe (OR = 0.55; 95 % CI: 0.35, 0.86) and moderate (OR = 0.82; 95 % CI: 0.69, 0.97) language delay in the children at age 3 years, but there was no association with gross motor skills [32]. In the third, children of mothers who used supplements scored ~4-5 points (0.3 SD) higher in motor skills, verbal ability and verbal-executive function compared to children of non-users at age 4 years, but not in perceptive performance or memory [34], and had a lower incidence of omission (better attention) but not commission errors at age 11 years (incidence rate ratio: 0.80; 95 % CI: 0.64, 1.00) [33]. A small study (*N* = 96) in a high risk population, with no information about confounders, found no difference in cognitive scores between the supplemented group and the general population [35].

Risk of bias was high in two studies (score: 6 [27] and 11 [31]), low in two (score: 17 [32] and 20 [36]) and medium in the others (score: 13-16).

### Maternal vitamin B12 (Table 5)

Of seven studies (all observational) [23, 25, 26, 28–30, 37] three were conducted in developing countries [25, 26, 28]. The sample size varied from 108 to over 6,000 mother-offspring pairs. Loss to follow-up was 10-20 % in three studies [23, 26, 29] and high (~50 %) in two [30, 37].

The exposure was measured in different ways, and at different gestational ages. In three studies the exposure was plasma B12 concentrations [23, 25, 26], assessed during the third trimester in two [25, 26] and during the second and the third trimester in another (which also assessed holotranscobalamin concentrations) [23]. In four studies, the exposure was average daily dietary B12 intake (FFQ and/or supplement use, self-reported) during the peri-conceptional period or the first and/or the second or the third trimester [28–30, 37]. Trained assessors assessed cognitive function in all the studies using a similar test battery (BSID) in two [23, 28] and by different instruments in all others. Only one study reported whether outcome assessors were blinded to the exposure [28].

The findings were inconsistent. In a rural Indian population with a very high prevalence (~70 %) of vitamin B12 deficiency, the study was conducted in a very small selected sample of children of mothers with extreme (low and high) vitamin B12 status [25]. Children of mothers in the lowest decile of B12 concentrations (*N* = 49) performed poorly in tests of sustained attention (182 vs. 159 seconds) and short-term memory (4.3 vs. 4.4 digits) compared to children of mothers in the highest decile (*N* = 59) at age 9 years. There were no associations between maternal B12 deficiency and scores in tests of intelligence and visual recognition. In an urban Indian study in which ~40 % of mothers were deficient, there were no associations between maternal B12 concentrations (either deficiency or across the range) and children’s cognitive function at age 9-10 years [26]. In a small Canadian study (*n* = 154), in which only ~ 8 % of mothers were B12 deficient, there were no associations with the children’s language, cognitive and motor skills at age 1.5 years [23]. In a Mexican cohort, low maternal B12 intake (<2 μg/day) was associated with lower MDI, but not PDI score (-1.6 score points (~0.3 SD); 95 % CI: -2.8, -0.3) in the children at age 1-12 months [28]. In an American study, maternal B12 intake from food and supplements during the second, but not during the first trimester and peri-conceptional period, was inversely related to offspring receptive language (-0.4 score points (0.03 SD)/2.6 μg/day; 95 % CI: -0.8, -0.1), but not visuo-motor abilities, at age 3 years [29]. But in the same cohort at seven years, with ~50 % attrition, B12 intake during the first and the second trimester was unrelated to offspring receptive language, verbal and non-verbal intelligence [30]. In a large well-nourished UK sample, with ~50 % attrition, there was no association of maternal B12 intake with offspring IQ at age 8 years. However, there was a significant association, with a very small effect size, between maternal genetic variants linked to plasma vitamin-B12 and offspring IQ [37].

Risk of bias was medium in all seven studies (score: 13-16).

### Maternal iron (Table 6)

Of eight studies (6 observational [31, 38–42] and 2 trials, one in Australia [43] and another in China [44], three were conducted in developing countries [40, 42, 44]. The sample size varied from 63 to over 10,000 mother-offspring pairs. Loss to follow-up was ~30 % in two studies, including the Australian trial [38, 43], ~10-23 % in four studies, including the Chinese trial [39, 40, 42, 44], and unavailable in the remainder [31, 41].

The exposure varied in all six observational studies [31, 38–42]. One used intake of iron supplements, with/without other vitamins/minerals (dose not available), three months prior to pregnancy and/or three months after conception [31]. In this study women also used folate supplements (details presented in folate section). Three studies used serum ferritin and/or haemoglobin concentrations and/or anaemia assessed during the first or the second and/or the third trimester [38, 39, 42]. In one of them, 90 % of mothers consumed a diet rich in iron and took iron supplements (27 mg) daily in the third trimester [38]. The fifth study (in mothers exposed to prenatal methyl mercury) used total body iron stores assessed (based on the ratio of the serum transferrin receptor to serum ferritin) at enrolment [40]. The sixth used haemoglobin level assessed before 18 weeks and after 28 weeks in pregnancy and maternal genes linked to iron or haemoglobin concentrations [41].

In the Australian trial, pregnant mothers received iron supplements (20 mg/day) or placebo from 20 weeks gestation until delivery [43]. In the Chinese trial, pregnant mothers were supplemented daily with 400 μg of folic acid alone (control group), or 400 μg folic acid with 60 mg iron, or 400 μg folic acid with 30 mg iron and multiple micronutrients, from enrolment (<28 weeks of gestation) until delivery [44].

Cognitive instruments differed between studies. Cognitive function was assessed by trained researchers in all except one, in which school scores were self-reported at age 14 years and teacher-rated at age 16 years [39]. In three studies, including the Chinese trial, outcome assessors were unaware about the exposure [39, 40, 44]; in the remaining studies this information was unavailable.

Findings from these studies were fairly consistent. Both trials, and four of the six observational studies found no associations of maternal iron status with offspring cognitive function [31, 38, 40, 41, 43, 44]. This was despite a good sample size and/or follow-up rates in three studies [31, 41, 44]. The remaining two observational studies found positive associations between maternal iron status and offspring cognitive function [39, 42]. One large study in Finland (*N* = ~10,000), found a small increase (0.03-0.06 SD) in children’s school performance score at age 14 and 16 years for each 10 g/L increase in maternal haemoglobin concentrations during the ninth (but not third or seventh) month of gestation [39]. Children of non-anaemic mothers had 0.04 to 0.07 SD higher school performance scores than children of anaemic mothers. In the other study in Vietnam, infants of anaemic mothers scored lower (-11.6 points (-0.7 SD); 95 % CI: -23.0, -0.2) in BSID composite score at age 6 months than the infants of non-anaemic mothers [42]. Both studies adjusted for multiple confounders (Table 6) but did not adjust for maternal IQ, home environment or the child’s own iron status.

Risk of bias was high in one study (score: 11) [31], medium in three (score: 15-16) [38, 41, 42] and low in the others, including the trials (score: 18-19).

### Maternal carbohydrate/protein (Table 7)

The only study that we found was a trial conducted in rural Gambia [45]. The sample size was 1459 mother-offspring pairs. The children’s age at assessment was 16-22 years; 285 children were <18 years of age. Loss to follow-up was ~20 %.

Mothers in the intervention group received 2 biscuits/day fortified with protein-energy (providing 1015 kcal energy and 22 g protein/day) from 20 weeks gestation until delivery.

Mothers in the control group received the same supplements for 20 weeks postpartum, but not during pregnancy. Cognitive domains were assessed using standard tests by trained examiners who were unaware of the exposure. Additionally, information about school achievement was obtained by questionnaire. Information about compliance was not reported. This trial showed no difference in cognitive test scores and school achievement between the prenatally supplemented group and controls, either unadjusted or after adjustment for confounders. Risk of bias was low (score: 19).

Since the exposures and cognitive tests varied between studies, we were unable to perform a meta-analysis for any of the nutrients.

## Discussion

In this systematic review of observational studies and trials covering maternal body mass index and single micronutrient effects, we explored evidence for a causal link between maternal nutritional status during pregnancy and offspring cognitive function during childhood and adolescence. There were very few studies from developing countries, where maternal nutritional deficits are most common. Low maternal BMI has been inadequately studied. There was consistent evidence (all observational) that maternal obesity is associated with lower cognitive function in children. Two out of three studies of maternal vitamin D status showed lower cognitive function in children of deficient mothers. One trial of folic acid supplementation showed no effects on the children’s cognitive function and evidence from 13 observational studies using blood levels, supplement use or dietary intake, was mixed. Among seven studies of vitamin B12, all observational, most showed no association with the children’s cognitive function, though two studies in highly deficient populations suggested a possible effect. Four out of six observational studies and two trials found no association of maternal iron status with offspring cognitive function. A trial of maternal carbohydrate/protein supplementation during pregnancy showed no association with offspring cognitive function. Since positive findings were mainly in observational studies, residual confounding is a concern and limits conclusions.

### Strengths and limitations

The review was conducted following CRD recommendations [5] and PRISMA guidelines [6].

Quality assessment was done by two independent reviewers. Most studies had a medium risk of bias and only 2 had a high risk. Exclusion of non-English language literature may have resulted in some important studies being missed. Although some studies with null findings were published (probably because of increased recent interest in this topic), publication bias is another potential limitation. We could not perform meta-analyses due to methodological differences in the published research. We did not include multiple micronutrient trials as there were already two recently published systematic reviews on this topic [3, 4], or trials of fatty acid supplementation which have also been systematically reviewed.

### Maternal anthropometry

Nine of the ten studies, all from developed countries showed an association of high maternal BMI with poorer cognitive function in the children [8–13, 15–17]. The findings are consistent with earlier systematic reviews [2, 46]. Since then three more studies have been published [13, 15, 17]. Although the threshold BMI at which the effect was observed varied between studies, there was evidence of a dose response effect in the majority. For example, compared to the normal-weight category the effect was significant in the extremely obese [11] or obese categories, but not in the overweight category [8–10, 15–17] or overweight and obese categories combined [12, 13]. The effect size was generally small, ~0.1-0.2 SD lower IQ/cognitive test scores in children of obese rather than normal mothers, and similar in most of the studies [10–12, 17]. Most of the studies found the effect for one or more of the mental development domains [10, 12, 13, 17]. In some the effect was found for mental development but not for motor development [8, 11, 15, 16]. Only one study found no association between maternal overweight and offspring cognitive function at age 2-3 years [14], possibly due to the young age at assessment (<3 years); most psychologists think 4 years is probably the earliest age for reliable estimates of cognitive function [47].

The association of maternal adiposity with reduced offspring cognitive function could be due to trans-placental transfer of inflammatory factors from maternal adipose tissue to the fetus [48, 49]. These inflammatory factors, which cross the blood brain barrier, could lead to inflammation of the brain, a reduction in fetal neurotrophic factors, and adversely affect neuronal differentiation, plasticity and function. Rodent studies in which obesity has been induced during pregnancy using high fat diets, have demonstrated increased inflammatory cytokines, lower levels of brain-derived neurotrophic factor (BDNF) in the offspring brain and poorer learning [48, 49]. Such experiments in humans are clearly impossible.

Confounding is another possible explanation for the findings. This is especially important in studies of obesity and cognitive function, both of which are strongly influenced by SES. Despite adjustment for SES, residual confounding could not be ruled out, since SES variables were often limited to fairly crude measures like income or occupation. Furthermore, in developed countries cohort studies have shown that lower IQ during childhood is linked with higher BMI/obesity in adulthood [50, 51]. Lack of adjustment for maternal intelligence (MIQ) could mean that any link between higher maternal BMI/obesity and offspring cognitive function was due to confounding. Only three of the nine studies that showed an association adjusted for MIQ [8, 10, 13].

Five studies from developed countries showed an association of low BMI with lower cognitive scores in the children, with a difference of 0.01-0.3 SD between children of underweight and normal-weight mothers [8, 10–12, 17]. But these differences were mainly non-significant, possibly due to lack of power, as the underweight category tended to be small. However, in two studies and in one of the two cohorts in another study there was a significantly higher risk of delayed mental development (risk ratio = 1.36) or lower IQ scores or mild intellectual disability (OR = 2.1) in children of underweight mothers [9, 11, 17]. Fetal exposure to nutrient deficiencies might lead to alterations in the neurotransmitter and neuroendocrine systems, and structural brain development [1, 52–56] and subsequent reduced cognitive function. Since the home environment, parental care and stimulation, in addition to socio-economic factors, influence cognitive function, inadequate adjustment for these factors could leave residual confounding.

Of the six studies that examined gestational weight gain as the exposure, all from developed populations, three found associations of reduced cognitive function which differed according to maternal weight status. Less than expected weight gain in normal weight mothers and more than expected weight gain in normal or already obese mothers was associated with lower offspring cognitive function [17–19]. This could indicate a causal association of maternal undernutrition or overnutrition with poorer offspring cognitive function for the reasons explained above. However the findings could also be due to confounding for the above explained reasons.

### Maternal vitamin D

Two of the three available studies, all from developed populations, showed an association of higher maternal vitamin D concentrations with better cognitive function in the children [21, 22]. Both showed evidence of a dose–response relationship. The effect size was modest. For example children of mothers with normal vitamin D status scored 0.1-0.2 SD higher in mental and psychomotor development tests compared to children of deficient mothers [22]. However, the percentage with language impairment was double in children of vitamin D deficient mothers compared to normal mothers in one study [21]. The effect was specific to language impairment in one study [21] and in the other effects were found for both mental and psychomotor development [22]. These findings are consistent with animal studies which have demonstrated poor learning and memory, and alterations in attention, in response to vitamin D deficiency before conception and/or during gestation [57, 58]. The findings are plausible due to a variety of biological actions of Vitamin D fundamental to neurodevelopment, including a signalling role in cell differentiation and synaptic formation [59], gene expression [59], regulation of the metabolism of neurotrophic and neurotoxic factors [60] and a protective role during brain inflammation [61]. Although both studies adjusted for confounders, socio-economic variables were limited to income or occupation. Another limitation was that maternal vitamin D status was available only during the second trimester; since vitamin D is known to fluctuate with sunlight exposure, the timing of deficiency may be crucial in determining cognitive function. A lack of trial data and a lack of studies from developing countries were notable omissions in the literature. Thus, based on a limited number of observational studies evidence linking maternal vitamin D deficiency with reduced offspring cognitive function is not conclusive.

### Maternal folate

Among the 14 studies reviewed, the findings were mixed. Of the 13 observational studies, mainly from developed populations, seven cohort studies and a case–control study in an African population showed positive associations of maternal folate (plasma concentrations or dietary intake or supplement use) with offspring cognitive function [26–29, 31–34]. Specificity varied between the studies. For example, in one there were associations with both mental and psychomotor development [34], while in another it was specific to mental development especially in children of mothers who were carriers of MTHFR677 TT genotype [28] and in others it was found for one or more of motor or mental development domains [26, 29, 31–33]. One study showed evidence of a dose–response relationship [26]; in others it was impossible to evaluate dose–response effects. Among the positive studies, the effect was quite large. For example, children of mothers who used folic acid supplements scored ~0.3 SD higher in mental and psychomotor development scores than non-users [34]. Children of mothers with lower dietary folate intake (<400 μg/day) scored 0.3 SD lower in MDI compared to children of mothers with adequate folate intake [28].

Folate plays a role in a number of biological actions that could influence neurodevelopment, such as myelination, and maintenance of tissue levels of neurotrophic and neurotoxic cytokines [62, 63]. However, confounding is a concern. Higher dietary intake and/or use of supplements may be an indicator of higher SES or higher MIQ and/or education. Although, the majority of the studies adjusted for confounders, adjustment was usually limited to income, occupation or education. Using self-reported exposure as a measure of nutrient status is another concern. As the majority of studies used exposures measured by self-reported questionnaires, there may be measurement error due to either under- or over reporting. This could introduce bias and limit interpretation. Other limitations of these studies included, parent-reported outcomes, potential observer bias and selective reporting. Furthermore, information about MIQ, home environment, level of adherence among supplement users and the child’s current folate status were generally unavailable. Most of the null studies had low power due to a small sample [23, 25, 35] and sample selection was of concern. For example, mothers had little variation in folate status in one study [24] and no folate deficiency in another [23]. A double blind randomised controlled trial overcomes many of these methodological issues, and the European trial was negative [36]. The trial, though adequately powered, was of reasonable quality, but it had high attrition rates, and compliance was not reported. Maternal folate status was not reported, and a trial in Europe, where women are likely to be relatively folate replete, does not rule out an effect in populations with high rates of folate deficiency.

### Maternal vitamin B12

The seven studies had inconsistent findings. Two of them, both from developing countries (India and Mexico) with high rates of B12 deficiency, found reduced cognitive function in children of deficient mothers or those with low dietary intakes [25, 28]. It was impossible to evaluate dose–response effects. Where present, there was quite a large effect. For example, compared to children of mothers with adequate dietary B12 intake children of mothers with lower B12 intake (<2 μg/day) scored 0.3 SD lower in MDI [28]. The effect was found for one or more mental development domains. The findings may indicate a biological relationship (mechanisms are similar to those of folate reported above). However, residual confounding remains a concern.

Of the remaining five studies, mostly in developed countries, two, conducted in the same cohort at different ages showed an association between maternal vitamin B12 dietary intake and offspring cognition at age 3 years [29] but not at age 7 years [30]. In the remaining three there was no evidence of an association [23, 25, 37]. This could be due to a young age at assessment (<2 years), a small sample, or insufficient variation in B12 status [23]; and the use of self-reported FFQ-based dietary B12 intake [37].

### Maternal iron

Of the eight studies, two observational studies, one in a developed [39] and another in a developing country [42], found an association between maternal haemoglobin concentrations/anaemia and offspring school performance score [39] or infant cognitive development [42]. The effect size varied, with a difference of 0.04-0.7 SD between the children of non-anaemic and anaemic mothers. The remaining studies, including two trials, found no associations of maternal iron (supplements/haemoglobin/ferritin levels) status with offspring cognitive function [31, 38, 40, 41, 43, 44]. Iron is required for cell differentiation, myelination and neurotransmitter synthesis, and could thus influence neurodevelopment [64]. Iron uptake by the brain is high during the third trimester of gestation, corresponding to the peak of myelinogenesis. However, our review of the available data provides little support for an effect of maternal iron status at this time. This might be due to methodological limitations. For example, in one study maternal supplement use was based on self prescription, the dose of the supplement was unavailable, maternal reported outcome data, and cognitive tests were designed to screen for developmental delay rather than to capture variations within the normal range [31]. In two studies, the sample was small and the children were young (6 months-to- < 3 years) [38, 40]. In one of them and in another large well conducted study there was little variation in maternal iron status [38, 41]. Neither of the trials showed an effect of iron supplementation on the children’s cognitive function, providing even stronger evidence for a lack of effect. The low dose of iron in the Australian trial [41], and high losses to follow-up in the Chinese trial [42] mean that there is still a need for more high-quality trial-based evidence, especially in iron-deficient populations.

### Maternal carbohydrate/protein

In the only study, a trial, generally well conducted but with no information about compliance, there was no evidence of benefit of maternal carbohydrate/protein supplementation on offspring cognition [45]. In a rural Gambian population where women are likely to be malnourished, the dose of the supplement might not be adequate enough to have a long-term benefit; however this trial showed a large benefit for birthweight and infant survival. There is a need for more trials to evaluate the benefit of maternal carbohydrate/protein intake on offspring cognitive function.

### Limitations of evidence

The primary and most important limitation was the small number of studies from developing countries. This is very important for several reasons. Nutritional deficiencies tend to be more common and more extreme in developing than in developed countries due to poverty and poor diets. The confounding structure in developing countries is often different from developed countries. In developing countries the burden of poor SES, low literacy and unemployment are more severe than in developed countries; the socio-cultural environment or dietary practices vary between developed and developing populations. Thus the impact of these factors in predicting maternal nutritional status and cognitive function are likely to differ from developed populations. Studies in developing countries may reveal associations between maternal nutritional status and children’s cognitive function that are not detectable in developed populations. A lack of experimental evidence, measurement error due to self-reported exposures and a young age at outcome assessment were major limitations. Only four out of the 34 observational studies and three out of the four trials reported a power calculation, and there is a need for better reporting in the literature on the adequacy of sample sizes. Although the quality score was medium in the majority of studies, some reported sample selection, attrition, power and observer bias inadequately, thus limiting the conclusions that can be drawn.

Experimental studies would provide better evidence. However, such studies are expensive and ethical issues are an important barrier. Iron and folic acid supplementation in pregnancy is now standard in most countries. As already described, a recent systematic review evaluated six multiple micronutrient trials, all conducted in low-income settings, to assess evidence linking maternal multiple micronutrient supplementation and offspring cognitive function. Three of them showed that maternal multiple micronutrient supplementation during pregnancy was beneficial for offspring cognitive function. The authors concluded that the evidence was inconclusive due to transient findings, methodological limitations and inadequate reporting and suggested further research [4]. Three more multiple micronutrient trials, all conducted in developing populations, have been published following this review [65–67]. Again, the findings were inconsistent and inconclusive. A study in Nepal [65] showed no associations between maternal multiple micronutrient supplementation during pregnancy and intellectual functioning in the offspring at age 7-9 years. Another in Indonesia showed a beneficial effect of maternal multiple micronutrient supplementation during pregnancy on children’s motor and attention/spatial ability at age 3.5 years only in the children of undernourished or anaemic mothers [66]. The third study in Viet Nam showed no associations between twice weekly maternal multiple micronutrient supplementation during pregnancy and offspring cognitive development at age 6 months [67].

Other than vitamins and minerals, fatty acids are another important and essential micronutrient required for optimal neurodevelopment and function. Findings from some observational and experimental studies suggest a beneficial association between maternal fatty acids status during pregnancy and offspring cognitive function. However, recommending routine supplementation of fatty acids and the amount required still remains a topic of debate [68].

There is debate about recommending multiple micronutrient supplementation as a routine instead of iron and folic acid, based on birthweight effects. It could be argued that if all the micronutrients are being supplemented there is no need to study effects of single micronutrient. However, a better understanding of which specific nutrients are important for neurodevelopment, and specific requirements in different settings and populations is required. There has also been concern that there are adverse interactions between micronutrients when supplied in a single preparation [69].

## Conclusions

Interest in the area of maternal nutrition and offspring cognitive function has increased in recent years. It is evident from this review that most of the studies were published in the last decade. We found some evidence linking maternal obesity and low micronutrient status, in particular, that of vitamin D, folate and B12 during pregnancy with poorer offspring cognitive function, suggesting that maternal nutrition is important for optimal offspring neurodevelopment and long-term cognition. However, a lack of data from developing populations and a lack of trial data limit conclusions. We suggest that there is a need for more experimental research in this area especially from developing countries.

## Abbreviations

BDNF, brain-derived neurotrophic factor; BMI, body mass index; BSID, Bayley Scale of Infant Development; CRD, Centre for Reviews and Dissemination; FFQ, food frequency questionnaire; IQ, intelligence quotient; MDI, mental development index; MeSH, medical subject headings; MIQ, m,aternal intelligence; MTHFR, methylenetetrahydrofolate reductase; PDI, psychomotor development index; PRISMA, Preferred Reporting Items for Systematic Reviews and Meta-Analyses; RCT, randomised controlled trial; SES, socio-economic status; WISC, Wechsler Intelligence Scales for Children

## Additional file

Additional file 1: Quality assessment form for a systematic review. (DOC 79 kb)
