# Supplementary material for: Association between maternal nutritional status in pregnancy and offspring cognitive function during childhood and adolescence; a systematic review
Source: BMC Pregnancy Childbirth. 2016 Aug 12;16:220. doi: 10.1186/s12884-016-1011-z (PMC4982007; doi:10.1186/s12884-016-1011-z)
Supplement: Additional file 1: — Quality assessment form for a systematic review. (DOC 79 kb) [file 12884_2016_1011_MOESM1_ESM.doc]

# Additional file 1 Quality assessment form for a systematic review

**Article ID: Author and Year of publication: Reviewer code:**

Article Title:

| **Quality** | **Criteria** | **Score** |
| --- | --- | --- |
| **Report** | 1. Is the hypothesis/aim/objective of the study clearly described?  **0-No; 1-Yes** |  |
|  | 2. Are the main outcomes to be measured clearly described in the introduction or methods section? **0-No; 1- Yes** |  |
|  | 3. Are the characteristics of the patients included in the study clearly described**? 0-No; 1- Yes** |  |
|  | 4. Are the main exposures to be measured clearly described in the introduction or methods section? **0-No; 1- Yes** |  |
|  | 5. Are the main ﬁndings of the study clearly described? **0-No; 1- Yes** |  |
|  | 6. Does the study provide estimates of the random variability in the data for the main outcomes? **0-No; 1- Yes** |  |
|  | 7. Have the number of patients lost to follow-up been given? **0-No; 1- Yes** |  |
|  | 8. Have actual probability values been reported (e.g. 0.035 rather than <0.05) for the main outcomes except where the probability value is less than 0.001? **0-No; 1- Yes** |  |
| **External validity** | 9. Were the subjects asked to participate in the study representative of the entire population from which they were recruited?  **0-No; 0-Unable to determine; 1-Yes** |  |
|  | 10. Were the subjects who were prepared to take part representative of the entire population from which they were recruited?  **0-No; 0-Unable to determine; 1-Yes** |  |
| **Internal validity** | 11. Was an attempt made to blind the assessor of the outcome about the main exposure? **0-No; 0-Unable to determine; 1-Yes** |  |
|  | 12. If any of the results of the study were based on “data dredging”, was this made clear? **0-No; 0-Unable to determine; 1-Yes** |  |
|  | 13. Do the analyses adjust for different lengths of follow-up of subjects?  **0-No; 0-Unable to determine; 1-Yes** |  |
|  | 14. Were the statistical tests used to assess the main outcomes appropriate? **0-No; 0-Unable to determine; 1-Yes** |  |
|  | 15. Were the main exposure measures used accurate (valid and reliable; recall bias)? **0-No; 0-Unable to determine; 1-Yes** |  |
|  | 16. Were the main outcome measures used accurate (valid and reliable; recall bias)? **0-No; 0-Unable to determine; 1-Yes** |  |
| **Internal validity confounding (selection bias)** | 17. Were the subjects in different exposure groups recruited from the same population? **0-No; 0-Unable to determine; 1-Yes** |  |
|  | 18. Were study subjects in different exposure groups recruited over the same period of time? **0-No; 0-Unable to determine; 1-Yes** |  |
|  | 19. Was there adequate adjustment for confounding in the analyses from which the main ﬁndings were drawn?  **0-No; 0-Unable to determine; 1-Yes** |  |
|  | 20. Were losses of subjects to follow-up taken into account?  **0-No; 0-Unable to determine 1-Yes** |  |
| **Power** | 21. Was the power calculation done before the study to detect a clinically important effect where the probability value for a difference being due to chance is less than 5%? **0-No; 0-Unable to determine 1-Yes** |  |
| **Conflict of interest** | 22. Was there a declaration of conflict of interest or identification of funding source **0-No; 1-Yes** |  |
|  | **Total score** |  |
